# Association of trauma classifications to long-term outcome in blunt trauma patients

**DOI:** 10.1007/s00068-024-02606-8

**Published:** 2024-08-07

**Authors:** Joonas Kuorikoski, Mikko Heinänen, Tuomas Brinck, Tim Söderlund

**Affiliations:** 1 Department of Orthopaedics and Traumatology, Tampere University Hospital, Tampere, Finland; 2 Trauma Unit, HUH Musculoskeletal and Plastic Surgery, Helsinki University Hospital, P.O. Box 266, Helsinki, 00029 HUS Finland; 3 Helsinki University and Helsinki University Hospital, Helsinki, Finland; 4 Department of Orthopaedics and Traumatology, Mehiläinen Hospital, Helsinki, Finland

**Keywords:** Polytrauma, Trauma, Trauma care, Berlin definition, Mortality, Trauma registry

## Abstract

**Purpose:**

The impact of major trauma is long lasting. Although polytrauma patients are currently identified with the Berlin polytrauma criteria, data on long-term outcomes are not available. In this study, we evaluated the association of trauma classification with long-term outcome in blunt-trauma patients.

**Methods:**

A trauma registry of a level I trauma centre was used for patient identification from 1.1.2006 to 31.12.2015. Patients were grouped as follows: (1) all severely injured trauma patients; (2) all severely injured polytrauma patients; 2a) severely injured patients with AIS ≥ 3 on two different body regions (Berlin-); 2b) severely injured patients with polytrauma and a physiological criterion (Berlin+); and (3) a non-polytrauma group. Kaplan-Meier survival analysis was performed to estimate differences in mortality between different groups.

**Results:**

We identified 3359 trauma patients for this study. Non-polytrauma was the largest group (2380 [70.9%] patients). A total of 500 (14.9%) patients fulfilled the criteria for Berlin + definition, leaving 479 (14.3%) polytrauma patients in Berlin- group. Berlin + patients had the highest short-term mortality compared with other groups, although the difference in cumulative mortality gradually plateaued compared with the non-polytrauma patient group; at the end of the 10-year follow up, the non-polytrauma group had the greatest mortality due to the high number of patients with traumatic brain injury (TBI).

**Conclusion:**

Excess mortality of polytrauma patients by Berlin definition occurs in the early phase (30-day mortality) and late deaths are rare. TBI causes high early mortality followed by increased long-term mortality.

## Introduction

Injury severity scoring began in the 1960s with organ-specific scoring with the Abbreviated Injury Scale (AIS) [1, 2]. The AIS scale has been updated since [3]. Trauma scoring has subsequently moved towards whole-body orientated injury scaling, such as the Injury Severity Score (ISS) [4] and New Injury Severity Score (NISS) [5]. Injury severity scores have facilitated comparison between different trauma patients and helped to explain and investigate differences in mortality between different hospitals [6]. Several systems (such as Trauma and Injury Severity Score [TRISS] [7, 8], Revised Injury Severity Classification [RISC] [9] and its update [RISCII] [10]) have been developed to evaluate expected 30-day mortality.

For decades there were multiple definitions of a polytrauma patient, and no validated definition existed [11]. A definition for polytrauma as AIS > 2 in two or more body regions was suggested in 2009 by Butcher and Balogh [12]. Based on this framework and the introduction of organized trauma systems, an international consensus meeting in 2014 in Berlin proposed a “Berlin definition” criteria for polytrauma patients (Table 1) [13].

**Table 1:** Berlin definition for polytrauma patient [13]

Always	AIS > 2 in two or more body regions	
	AND	
Any of these	At least one of the following physiologic parameters must exist:	
	Hypotension (systolic blood pressure ≤ 90 mmHg)	
	Level of consciousness (GCS score ≤ 8)	
	Acidosis (base excess ≤ -6.0)	
	Coagulopathy (INR ≥ 1.4/partial thromboplastin time ≥ 40 s)	
	Age (≥ 70 years)	

AIS > 2 AIS, Abbreviated Injury Scale; GCS, Glasgow coma scale; INR, international normalized ratio

Utstein criteria, uniform and standardized inclusion and exclusion criteria of trauma registries, and a minimum list of core data variables with precise definitions recommend short-term mortality as a trauma outcome measure, which appears sufficient [14–16]. However, the impact of a major trauma on mortality is noted to last much longer than 1 month after trauma [17–22].

In this study, we sought to evaluate the differences in long-term mortality beyond Utstein’s 30-day criteria of different trauma classifications, including all severely injured trauma patients (NISS > 15), severely injured patients with AIS ≥ 3 on two different body regions (polytrauma), polytrauma patients by Berlin definition (polytrauma and a physiological criteria), and non-polytrauma patients.

## Materials and methods

The Helsinki University Hospital (HUH) Trauma Unit is the only a level I trauma centre in Southern Finland and is responsible for treatment of all major trauma patients within its catchment area. The catchment area of HUH includes approximately 1.8 million inhabitants, making it one of the largest trauma centres in Northern Europe. The Helsinki Trauma Registry (HTR) is the trauma registry of the HUH Trauma Unit.

HTR was used for patient identification from 1.1.2006 to 31.12.2015. The HTR includes only patients treated at the HUH Trauma Unit. The inclusion criteria for the HTR were ISS ≥ 16 (2006 to 2011) or NISS ≥ 16 (from 2012 onwards) and treatment was required to start within 24 h after the accident. Children < 16 years are not included in the registry, as they are treated at another unit of the same university hospital. However, paediatric major trauma patients with head injury or suspected head injury were included in the HTR until 1.9.2015. In this study, we included patients with NISS ≥ 16 and blunt injury mechanism. Spinal-cord injuries were excluded to have more comparable data, as spinal-cord injury causes a long lasting major effect on patients health. We defined severe traumatic brain injury (TBI) as head AIS > 2, which has been defined as a cutoff value for a moderate TBI [23].

For analysis, patients were divided into five groups as follows. The first group included all severely injured trauma patients (All patients) and the second group included all severely injured polytrauma patients with AIS ≥ 3 on at least two different body regions (Polytrauma). This group was further divided into the following two subgroups: severely injured polytrauma patients with polytrauma **and** ≥ 1 physiological criteria (Berlin+) and those without physiological criteria (Berlin-). The fifth group included patients with an isolated severe injury, meaning patients who had either injuries in an isolated body region according to AIS [1] or only one AIS ≥ 3 injury and the remaining injuries AIS ≤ 2 (non-polytrauma group).

Survival data were obtained from the Population Register Centre of Finland. The 10-year follow up concluded at death, at the end of the follow-up period, or on the date the person moved to another country.

We calculated average annual long-term mortality (i.e. number of patients deceased per year during the follow up) percentages for all groups with the following formula: (change in survival 30 d– 10 y) / 119 months * 12 months. This allowed us to exclude the effects of acute-phase mortality. We excluded isolated face and neck injuries from our analysis due to the low number of cases.

Different groups were identified, and Kaplan-Meier survival curves were calculated to estimate the differences in mortality between different groups. Log-rank and Kruskal-Wallis were used as appropriate.

To obtain more detailed information on the largest patient group (non-polytrauma, *n* = 2380), this group was further divided into different groups by AIS regions. Kaplan-Meier survival analysis was performed for the different groups.

**Fig. 1:**
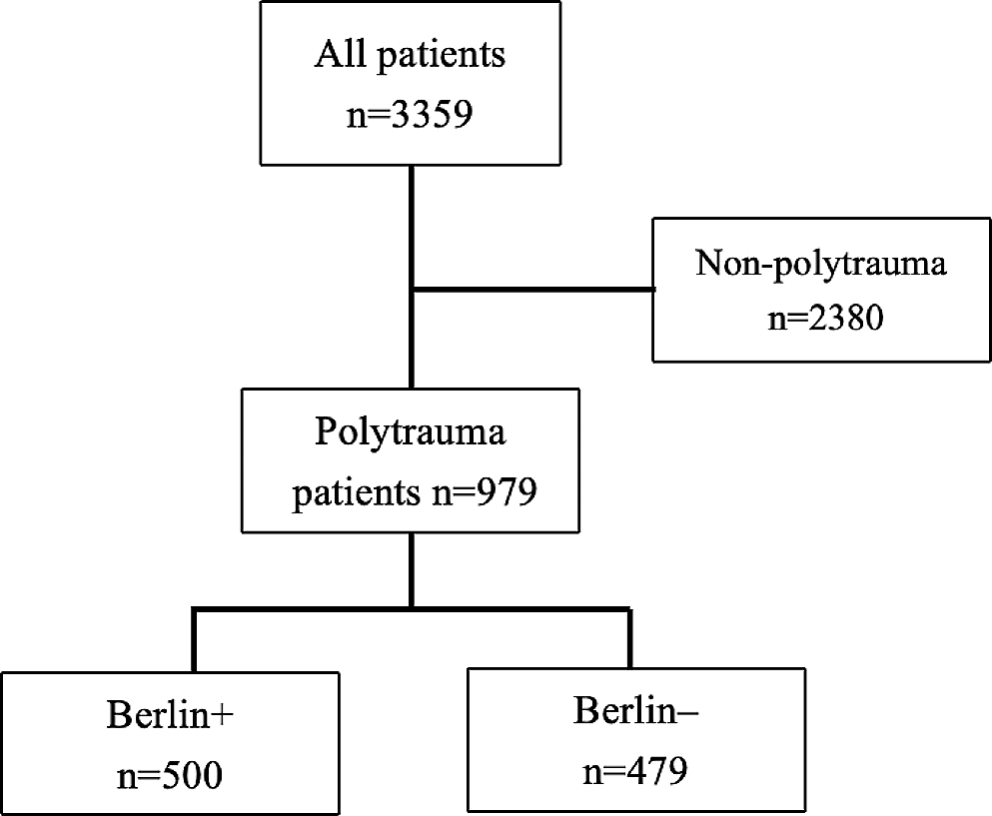
Classification flowchart of included trauma patients of different groups

IBM SPSS Statistics Version 27 and R Core Team (2022) R: A language and environment for statistical computing, R Foundation for Statistical Computing, Vienna, Austria (URL https://www.R-project.org/) were used for statistical analyses and graphs. Microsoft^®^ PowerPoint for Mac was used to create Fig. 1.

This was a retrospective register study. The HUH scientific review board accepted the study (HUS/221/2017). According to Finnish legislation, consent from patients is not needed for a register study of this type. Data for the register were gathered by patient medical chart review.

## Results

We identified 3557 patients from HTR between 1.1.2006 to 31.12.2015. One patient with stab injuries, two patients with burn injury mechanism, and 193 patients with spinal-cord injuries were excluded. A total of 3359 trauma patients were included in the final analyses. Mean follow-up time for all patients was 1547 days (SD 1164, range 0–3648 days).

Berlin (+), patients that fulfil Berlin definition; Berlin (-), polytrauma patients that do not fulfil the Berlin definition.

Non-polytrauma was the largest group (*n* = 2380, 70.9% patients). Polytrauma patients (*n* = 979, 29.1%) were further divided into two groups (Berlin + and Berlin-). A total of 500 (14.9%) patients fulfilled the criteria for Berlin definition of polytrauma Berlin+, leaving 479 (14.3%) polytrauma patients in the Berlin- group. Male gender dominated in all groups. Patients in the Berlin + and Berlin- groups were younger than patients in the non-polytrauma group. Patients in polytrauma groups had higher NISS scores, were ventilated longer, and had longer ICU or hospital length of stay (LOS) than patients in non-polytrauma group (Kruskal-Wallis *p* < 0.001). Detailed information on patient demographics and clinical characteristics is shown in Table 2.

**Table 2:** Patient demographics and clinical characteristics

		All cases	Polytrauma patients	Berlin+	Berlin-	Non-polytrauma
Count	n	3359	979	500	479	2380
Gender	Female	932 (27.7%)	258 (26.4%)	152 (30.4%)	106 (22.1%)	674 (28.3%)
	Male	2427 (72.3%)	721 (73.6%)	348 (69.6%)	373 (77.9%)	1706 (71.7%)
Age (y)	Mean ± SD	51 ± 20	43 ± 21	45 ± 23	40 ± 17	54 ± 20
	Median (IQ25,75)	53 (34,65)	42 (24,59)	44 (23,65)	41 (25,54)	56 (41,68)
NISS	Mean ± SD	31 ± 12	36 ± 13	41 ± 13	31 ± 9	29 ± 10
	Median (IQ25,75)	29 (24,38)	34 (27,43)	41 (29,50)	29 (27,34)	26 (22,34)
ICU	%	79	81	82	81	78
ICU (d)	Mean ± SD	7 ± 8	10 ± 9	12 ± 10	7 ± 7	5 ± 6
	Median (IQ25,75)	4 (2,9)	7 (3,13)	9 (5,16)	5 (3,10)	4 (2,7)
Ventilated (d)	Mean ± SD	4 ± 6	5 ± 8	6 ± 9	3 ± 5	3 ± 5
	Median (IQ25,75)	1 (0,5)	2 (0,7)	4 (0,10)	0 (0,4)	1 (0,4)
LOS (d)	Mean ± SD	12 ± 12	14 ± 13	15 ± 15	13 ± 10	11 ± 12
	Median (IQ25,75)	9 (4,15)	11 (6,18)	11 (4,21)	10 (6,16)	8 (4,14)
GCS	13–15	2214 (66.2%)	623 (63.7%)	196 (39.3%)	427 (89.1%)	1591 (67.2%)
	9–12	339 (10.1%)	85 (8.7%)	33 (6.6%)	52 (10.9%)	254 (10.7%)
	5–8	314 (9.4%)	94 (9.6%)	94 (18.8%)	0	220 (9.3%)
	3–4	477 (14.3%)	176 (18.0%)	176 (35.3%)	0	301 (12.7%)

NISS, New injury severity score; GCS, Glasgow Coma Score; AIS, abbreviated injury score; ICU, intensive care unit; LOS, length of stay in hospital, d, days; y, years; ICU (%), percentage of patients that were treated in the ICU

**Fig. 2:**
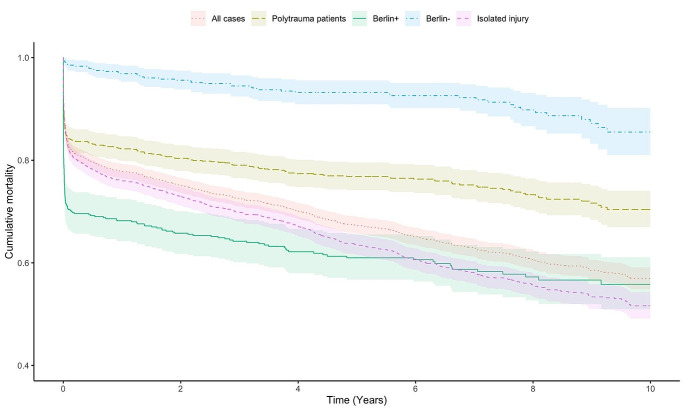
Kaplan-Meier survival graph of different patient groups

Berlin + patients had the highest short-term mortality compared with other groups. However, the difference in cumulative mortality gradually plateaued compared with the non-polytrauma patient group (i.e. at the end of the 10-year follow-up, the non-polytrauma group had the highest mortality; log-rank *p* < 0.001). Patients in Berlin- group had the lowest mortality throughout the observation period. Mortality analyses are shown in Fig. 2; Table 3.

**Table 3:** 30-day and 10-year mortality

	Survival	SD
30 days		
All patients	0.830	0.006
Polytrauma patients	0.842	0.012
Berlin+	0.704	0.020
Berlin-	0.985	0.005
Non-polytrauma	0.825	0.008
10 years		
All patients	0.569	0.011
Polytrauma patients	0.704	0.018
Berlin+	0.558	0.026
Berlin-	0.855	0.024
Non-polytrauma	0.516	0.013

Survival, proportion of survived patients; SD, standard deviation

Average annual long-term mortality was calculated for all groups. The annual mortality rates after 30 days of injury were as follows: all patients 2.6% (95%CI 2.5–2.8%), polytrauma patients 1.4% (95%CI 1.1–4.1%), Berlin + patients 1.5% (95%CI 1.0-1.9%), Berlin- patients 1.3% (95%CI 1.0-1.6%), and non-polytrauma group 3.1% (95%CI 2.9–3.1%).

Most of the included patients (*n* = 2254, 67%) had a TBI. TBI was present in 69% (*n* = 343) of the Berlin + group and 41% (*n* = 196) of the Berlin- group. The greatest proportion of patients with TBI was observed in the non-polytrauma group (72% (*n* = 1715).

**Fig. 3:**
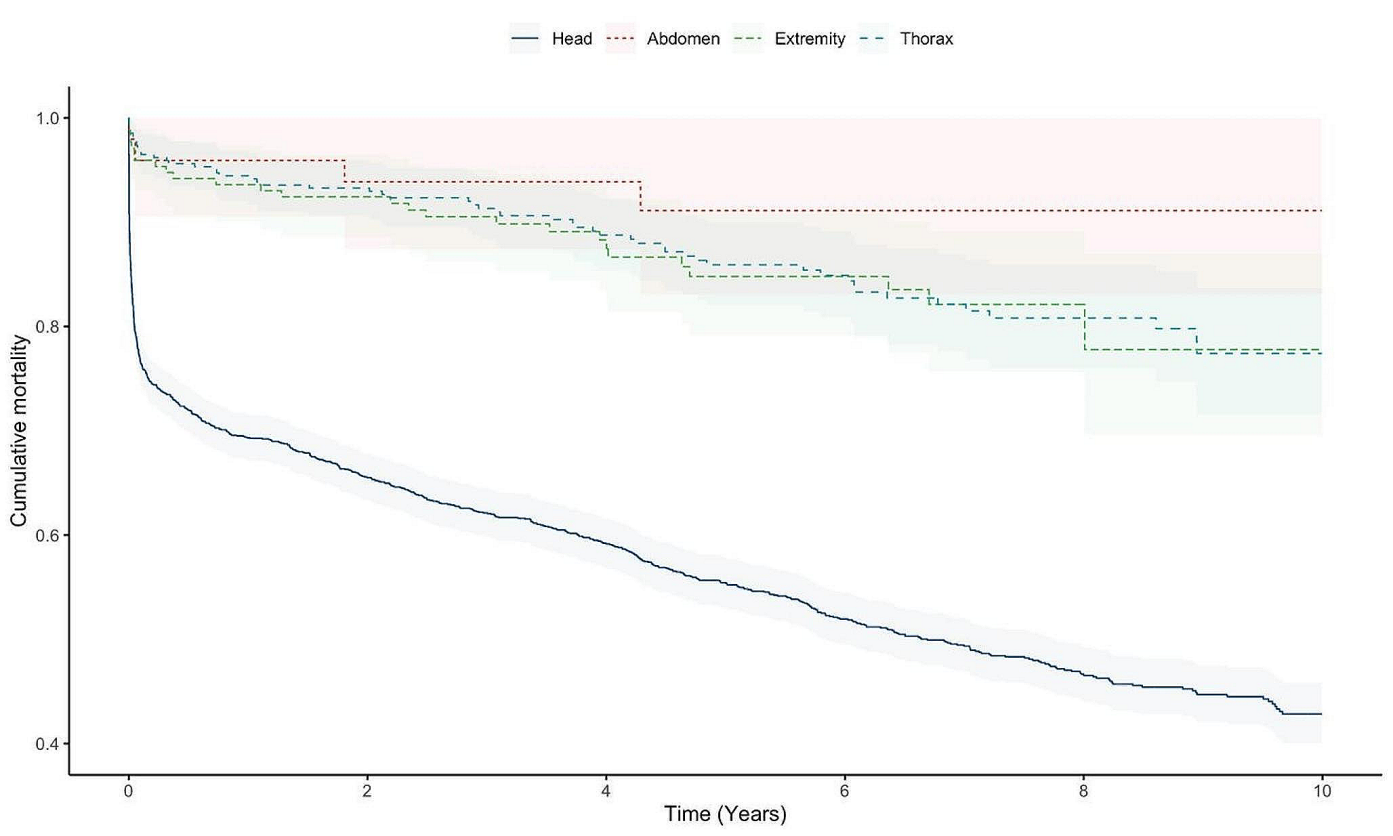
Kaplan-Meier graph of non-polytrauma group divided by different AIS regions

Further analysis of the non-polytrauma group revealed that the head AIS group (*n* = 1811, 76.1%) had significantly (log-rank *p* < 0.001) greater cumulative mortality than patients in other the AIS-region groups. Calculated annual mortalities for patients in the non-polytrauma groups were 0.5% (abdomen), 1.8% (extremities), 3.5% (head), and 2.0% (thorax). The neck AIS group had only two patients and was excluded from Kaplan-Meier Analysis (Table 4; Fig. 3).

**Table 4:** 30-day and 10-year mortality of patients

	Survival	SD
30 days		
Abdomen	0.959	0.028
Extremities	0.959	0.015
Head	0.781	0.010
Thorax	0.971	0.009
10 years		
Abdomen	0.911	0.043
Extremities	0.778	0.004
Head	0.438	0.015
Thorax	0.774	0.031

Survival, proportion of survived patients; SD, standard deviation

Mean age of the head AIS group was also the greatest among the non-polytrauma group. The head AIS group also had high 30-day and 10-year mortality (i.e. acute mortality was followed by elevated cumulative long-term mortality) (Table 5).

**Table 5:** Non-polytrauma group age and injury location

				Age ± SD
		n	%	Median (IQ25,75)
Head		1811	76.1	56 ± 19
				57 (45,69)
Face		4	0.2	59 ± 28
				69 (41,78)
Neck		2	0.1	45 ± 42
				45 (15,74)
Thorax		342	14.4	50 ± 19
				51 (35,63)
Abdomen		49	2.1	37 ± 20
				30 (22, 49)
Extremities		172	7.2	43 ± 21
				40 (23,62)
Total		2380	100	54 ± 20
				56 (41,68)

## Discussion

To our knowledge, this is the largest study that evaluated long-term mortality of severely injured patients divided into groups by different definitions. Polytrauma patients by Berlin definition had the greatest short-term mortality. Survival during the first 30 days after injury in these Berlin + patients suggests good long-term prognosis. However, Berlin- polytrauma patients have good short- and long-term prognosis. Patients with severe isolated injury should be evaluated by the body region affected rather than as a uniform group. Patients with isolated severe TBI have similar short-term mortality as Berlin + patients, but the long-term outcome is poorer in patients with isolated TBI.

Long-term mortality has been previously reported to remain high for the entire severely injured trauma patient group [17–22], and the same finding is particularly true for the non-polytrauma group with the highest annual long-term mortality percent. Interestingly, in the long term, Berlin + polytrauma patients have increased mortality only early after the injury. However, those surviving the acute phase have lower annual mortality than non-polytrauma patients. In a previous study of the same patient population, control subjects (10 controls/patient, matched for age, sex, and county of living) had a long-term annual mortality of 1.6% [21]. The annual mortality rate was comparable with control subjects in all groups except in isolated head injury, for which the annual mortality rate is over 2-fold greater (3.5%).

Increased mortality after sustaining a fracture has been previously observed [24, 25]. In the current study we identified an annual mortality of 1.8% in the extremities group of the non-polytrauma group. This is only marginally higher than the 1.6% annual mortality of the control group in a previous study [21]. High annual long-term mortality of the entire non-polytrauma group is explained by high annual mortality in the head group (3.5%) and in the thorax group (2.0%), followed by extremities (1.8%) and isolated injuries in abdomen (0.5%). Acute mortality in isolated head injuries is high and is further followed by high annual long-term mortality.

Patients in the non-polytrauma group were older than those in other groups. This may partly explain the better long-term prognosis of the other groups. The Berlin + group had higher mean age compared with the Berlin- group, probably because age is one of the five criteria in the Berlin definition.

The Berlin- group had the lowest cumulative mortality throughout the study period. Thus, the definition of polytrauma as AIS > 2 in two body regions contains two distinct groups based on their mortality. Berlin + and Berlin- groups have similar long-term annual mortality after the acute phase. The Berlin + group has high acute-phase (30-day) mortality (29.6%), whereas the acute mortality of the Berlin- group is low (1.5%).

Berlin + had the highest NISS scores, which are associated with higher short-term mortality [26]. However, patients in this group were younger than in the non-polytrauma group. Therefore, we can consider that patients in Berlin + group have good ability to recover from severe trauma if they survive the acute burden of the injury.

The proportion of TBI was moderately high in the Berlin + and Berlin- groups. TBI was seen in 72% of patients in the non-polytrauma group. Pfeifer et al. stated that CNS injury is one of the leading causes of death in trauma patients [27].

A weakness of this study is its retrospective nature. Strengths of this study include the large number of patients, the quality of the HTR [28], and the long follow-up period.

## Conclusion

Polytrauma patients by Berlin definition and patients with isolated TBI have high short-term mortality compared with other severely injured patients. However, in the long term the mortality difference diminishes in Berlin + patients. Thus, the excess mortality in Berlin + patients occurs in the early phase (30-day mortality) and late deaths are rare. In contrast, in isolated head injuries the increased acute mortality is followed by elevated long-term annual mortality. More detailed knowledge on the acute and long-term outcomes after severe injury is important for the patient, the patient’s relatives, and the healthcare system.

## Data Availability

Data are available on request provided that individual privacy is not compromised. Please contact author S.T for data queries.
